# Supplementary material for: Giant worm-shaped ESCRT scaffolds surround actin-independent integrin clusters
Source: J Cell Biol. 2023 May 18;222(7):e202205130. doi: 10.1083/jcb.202205130 (PMC10200693; doi:10.1083/jcb.202205130)
Supplement: Data S1 — shows the sequence of the IST1-GFP construct and of the Fiji macro for the analysis of the number and size of the ESCRT structures. [file JCB_202205130_DataS1.docx]

IST1-GFP construct

>IST1 without stopcodon:

Atgctg ggctctggat ttaaagctga gcgcttaaga gtgaatttga gattagtcat aaatcgcctt aaactattgg agaaaaagaa aacggaactg gcccagaaag caaggaagga gattgctgac tatctggctg ctgggaaaga tgaacgagct cggatccgtg tggagcacat tatccgggaa gactacctcg tggaggccat ggagatcctg gagctgtact gtgacctgct gctggctcgg tttggcctta tccagtctat gaaggaacta gattctggtc tggctgaatc tgtgtctaca ttgatctggg ctgctcctcg actccagtca gaagtggctg agttgaaaat agttgctgat cagctctgtg ccaagtatag caaggaatat ggcaagctat gtaggaccaa ccagattgga actgtgaatg acaggctaat gcacaagctg agtgtggaag ccccacccaa aatcctggtg gagagatacc tgattgaaat tgcaaagaat tacaacgtac cctatgaacc tgactctgtg gtcatggcag aagctcctcc tggggtagag acagatctta ttgatgttgg attcacagat gatgtgaaga aaggaggccc tggaagagga gggagtggtg gcttcacagc accagttggt ggacctgatg gaacggtgcc aatgcccatg cccatgccca tgcctatgcc atctgcaaat acgcctttct catatccact gccaaaggga ccatcagatt tcaatggact gccaatgggg acttatcagg cctttcccaa tattcatcca cctcagatac cagcaactcc cccatcgtat gaatctgtag atgacattaa tgctgataag aatatctctt ctgcacagat tgttggtcct ggacccaagc cagaagcctc tgcaaagctt ccttccagac ctgcagataa ctatgacaac tttgtcctac cagagttgcc atctgtgcca gacacactac caactgcatc tgctggtgcc agcacctcag catctgaaga cattgacttt gatgatcttt cccggaggtt tgaagagctg aaaaagaaaa ca

>Vector: EGFP-N1

>Restriction sites:

XHoI: CTCGAG
BamHI: GGATCC

>Linker: GGC GGC GGC GGC AGC

>Linker with first base removed to make IST1 gene in frame with EGFP in pEGFP-N1:

GC GGC GGC GGC AGC

>Kozak: GCC GCC ACC

>Codon-improved sequence (avoiding only XhoI and BamHI and NotI):

ATGCTGGGCAGCGGCTTCAAGGCCGAGCGCCTGCGCGTGAACCTGCGCCTGGTGATCAACCGCCTGAAGCTGCTGGAGAAGAAGAAGACCGAGCTGGCCCAGAAGGCCCGCAAGGAGATCGCCGACTACCTGGCCGCCGGCAAGGACGAGCGCGCCCGCATCCGCGTGGAGCACATCATCCGCGAGGACTACCTGGTGGA 200

GGCCATGGAGATCCTGGAGCTGTACTGCGACCTGCTGCTGGCCCGCTTCGGCCTGATCCAGAGCATGAAGGAGCTGGACAGCGGCCTGGCCGAGAGCGTGAGCACCCTGATCTGGGCCGCCCCCCGCCTGCAGAGCGAGGTGGCCGAGCTGAAGATCGTGGCCGACCAGCTGTGCGCCAAGTACAGCAAGGAGTACGGCA 400

AGCTGTGCCGCACCAACCAGATCGGCACCGTGAACGACCGCCTGATGCACAAGCTGAGCGTGGAGGCCCCCCCCAAGATCCTGGTGGAGCGCTACCTGATCGAGATCGCCAAGAACTACAACGTGCCCTACGAGCCCGACAGCGTGGTGATGGCCGAGGCCCCCCCCGGCGTGGAGACCGACCTGATCGACGTGGGCTTC 600

ACCGACGACGTGAAGAAGGGCGGCCCCGGCCGCGGCGGCAGCGGCGGCTTCACCGCCCCCGTGGGCGGCCCCGACGGCACCGTGCCCATGCCCATGCCCATGCCCATGCCCATGCCCAGCGCCAACACCCCCTTCAGCTACCCCCTGCCCAAGGGCCCCAGCGACTTCAACGGCCTGCCCATGGGCACCTACCAGGCCTT 800

CCCCAACATCCACCCCCCCCAGATCCCCGCCACCCCCCCCAGCTACGAGAGCGTGGACGACATCAACGCCGACAAGAACATCAGCAGCGCCCAGATCGTGGGCCCCGGCCCCAAGCCCGAGGCCAGCGCCAAGCTGCCCAGCCGCCCCGCCGACAACTACGACAACTTCGTGCTGCCCGAGCTGCCCAGCGTGCCCGACA 1000

CCCTGCCCACCGCCAGCGCCGGCGCCAGCACCAGCGCCAGCGAGGACATCGACTTCGACGACCTGAGCCGCCGCTTCGAGGAGCTGAAGAAGAAGACC

CAI-Value of the improved sequence:0.959142692834618
GC-Content of the improved sequence:68.48816029143899

>Complete insert (XhoI, Kozak, codon improved IST1, linker, BamHI):

CTCGAG

GCCGCCACC

ATGCTGGGCAGCGGCTTCAAGGCCGAGCGCCTGCGCGTGAACCTGCGCCTGGTGATCAACCGCCTGAAGCTGCTGGAGAAGAAGAAGACCGAGCTGGCCCAGAAGGCCCGCAAGGAGATCGCCGACTACCTGGCCGCCGGCAAGGACGAGCGCGCCCGCATCCGCGTGGAGCACATCATCCGCGAGGACTACCTGGTGGAGGCCATGGAGATCCTGGAGCTGTACTGCGACCTGCTGCTGGCCCGCTTCGGCCTGATCCAGAGCATGAAGGAGCTGGACAGCGGCCTGGCCGAGAGCGTGAGCACCCTGATCTGGGCCGCCCCCCGCCTGCAGAGCGAGGTGGCCGAGCTGAAGATCGTGGCCGACCAGCTGTGCGCCAAGTACAGCAAGGAGTACGGCAAGCTGTGCCGCACCAACCAGATCGGCACCGTGAACGACCGCCTGATGCACAAGCTGAGCGTGGAGGCCCCCCCCAAGATCCTGGTGGAGCGCTACCTGATCGAGATCGCCAAGAACTACAACGTGCCCTACGAGCCCGACAGCGTGGTGATGGCCGAGGCCCCCCCCGGCGTGGAGACCGACCTGATCGACGTGGGCTTCACCGACGACGTGAAGAAGGGCGGCCCCGGCCGCGGCGGCAGCGGCGGCTTCACCGCCCCCGTGGGCGGCCCCGACGGCACCGTGCCCATGCCCATGCCCATGCCCATGCCCATGCCCAGCGCCAACACCCCCTTCAGCTACCCCCTGCCCAAGGGCCCCAGCGACTTCAACGGCCTGCCCATGGGCACCTACCAGGCCTTCCCCAACATCCACCCCCCCCAGATCCCCGCCACCCCCCCCAGCTACGAGAGCGTGGACGACATCAACGCCGACAAGAACATCAGCAGCGCCCAGATCGTGGGCCCCGGCCCCAAGCCCGAGGCCAGCGCCAAGCTGCCCAGCCGCCCCGCCGACAACTACGACAACTTCGTGCTGCCCGAGCTGCCCAGCGTGCCCGACACCCTGCCCACCGCCAGCGCCGGCGCCAGCACCAGCGCCAGCGAGGACATCGACTTCGACGACCTGAGCCGCCGCTTCGAGGAGCTGAAGAAGAAGACC

GCG GCG GCG GCA GC

G GAT CC

Macro used for determining the number and size of the ESCRT structures

The channels are in the following order:
1: Phalloidin
2: Protein of choice
3: DAPI
4: IST1
5: Brightfield

*Creating region of interest (ROI) in the phalloidin channel*

setSlice(1);

run("Duplicate...", "duplicate channels=1");

setThreshold(500, 65535);

run("Convert to Mask");

*Set threshold for ESCRT-structures manually*

run("Select None");

run("Clear Results");

setSlice(4);

run("Duplicate...", "duplicate channels=4");

run("8-bit");

setMinAndMax(50,81);

setAutoThreshold("Default dark");

setThreshold(75, 255);

*Calculate number and size of the ESCRT-structures in the whole image*

run("Convert to Mask");

run("Select All");

run("Analyze Particles...", "size=0.30-Infinity display clear");

String.copyResults();

*Calculate number and size of the ESCRT-structures inside the cell (using ROI)*

run("Convert to Mask");

roiManager("Select", 0)

run("Analyze Particles...", "size=0.30-Infinity display clear");

String.copyResults();

*Calculate number and size of the ESCRT-structures outside the cell (using ROI)*

run("Convert to Mask");

roiManager("Select", 0)

run("Make Inverse");

run("Analyze Particles...", "size=0.30-Infinity display clear");

String.copyResults();
