# Supplementary figures and images for: Giant worm-shaped ESCRT scaffolds surround actin-independent integrin clusters
Source: J Cell Biol. 2023 May 18;222(7):e202205130. doi: 10.1083/jcb.202205130 (PMC10200693; doi:10.1083/jcb.202205130)

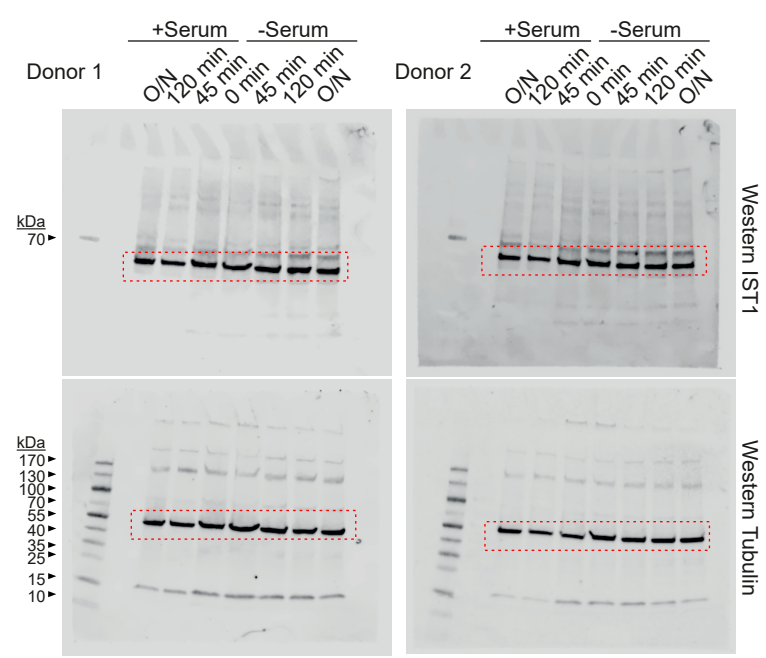

Supplement: SourceData FS1 — is the source file for Fig. S1. [file JCB_202205130_SourceDataFS1.pdf]

Figure S5A:

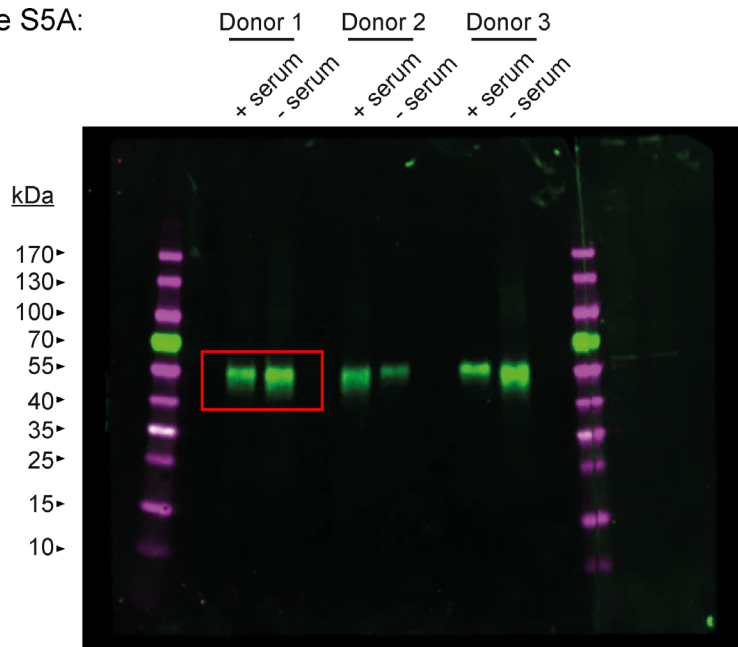

Figure S5B:

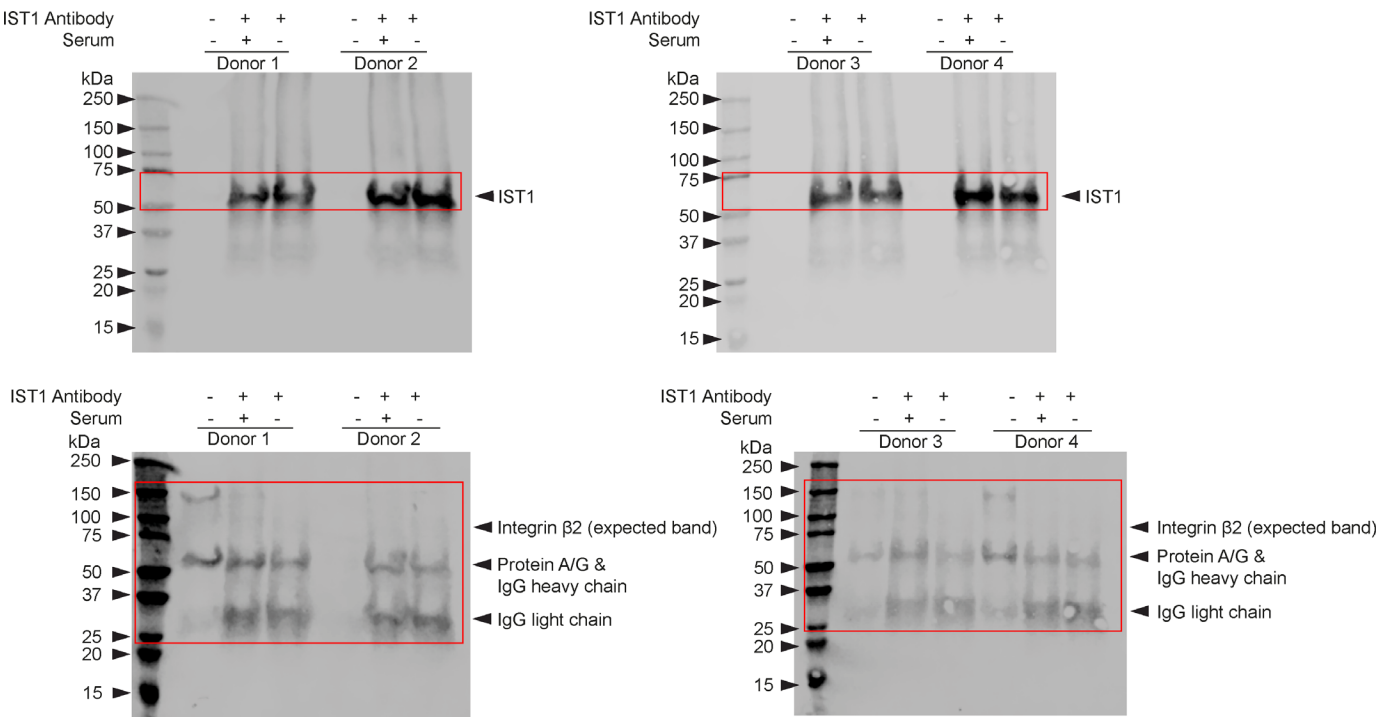

Supplement: SourceData FS5 — is the source file for Fig. S5. [file JCB_202205130_SourceDataFS5.pdf]

Figure S7C:

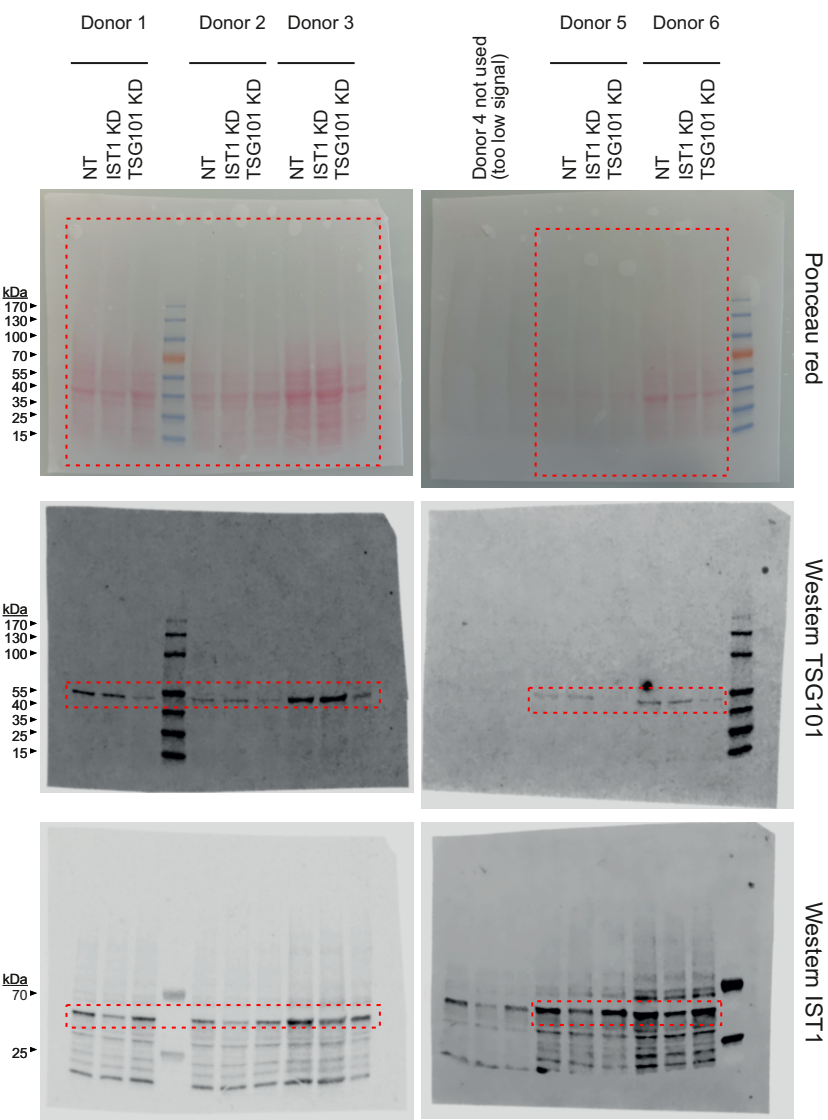

Figure S7G:

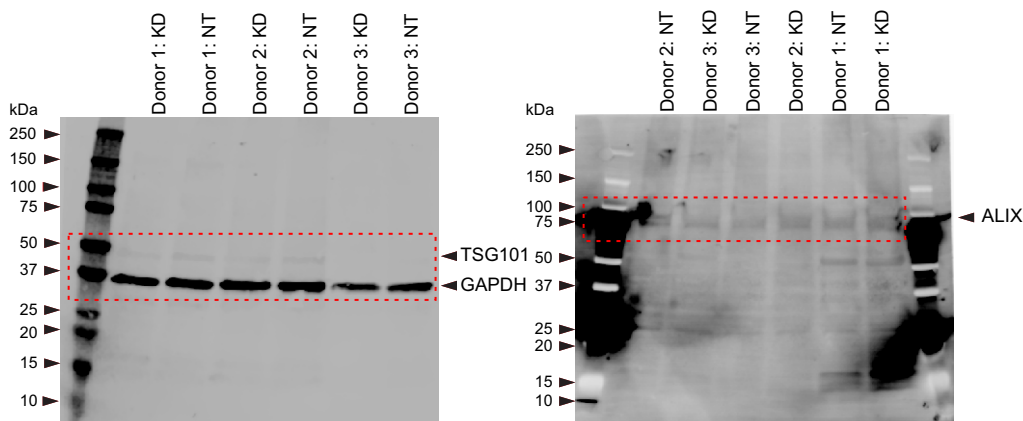

Supplement: SourceData FS7 — is the source file for Fig. S7. [file JCB_202205130_SourceDataFS7.pdf]
